# Transitions from pediatric to adult rheumatology care for juvenile idiopathic arthritis: a patient led qualitative study

**DOI:** 10.1186/s41927-022-00316-5

**Published:** 2022-11-14

**Authors:** Gillian R. Currie, M. Harris, L. McClinton, N. Trehan, A. Van Dusen, M. Shariff, T. Kuzmyn, D. A. Marshall

**Affiliations:** 1 Department of Community Health Sciences, Cumming School of Medicine, University of Calgary, 3280 Hospital Drive NW, Calgary, AB T2N 4Z6 Canada; 2 O’Brien Institute of Public Health, University of Calgary, Calgary, Canada; 3 Department of Pediatrics, University of Calgary, Calgary, Canada; 4 Alberta Children’s Hospital Research Institute, University of Calgary, Calgary, Canada; 5 McCaig Institute for Bone and Joint Health, University of Calgary, Calgary, Canada

**Keywords:** Juvenile idiopathic arthritis, Transition, Qualitative research, Focus groups, Patient-led research

## Abstract

**Background:**

Juvenile idiopathic arthritis (JIA) is a childhood autoimmune disease that causes swelling and pain in at least one joint. Young people with JIA experience symptoms that persist into adulthood, and thus will undergo a transition including the o transfer of care from a pediatric rheumatologist an adult rheumatologist. Missing from the literature is research that centres the transition experience of young people with JIA in Canada. This goal of this patient-led research was to explore the experience young people with JIA through the process of transition.

**Methods:**

Qualitative study using the Patient and Community Engaged Research (PaCER) approach. Trained patient-researchers conducted three focus groups using the Set, Collect and Reflect PaCER process. Participants, recruited via purposive and snowball sampling using research/personal networks and social media, were young people with JIA in Canada between 18 and 28 years who had experienced with the process of transition to adult care. Recordings were transcribed verbatim. Patient researchers individually coded overlapping sections of the data, and thematic analysis was conducted.

**Results:**

In total, nine individuals participated in one or more focus groups. Three themes were identified, with sub-themes: preparedness for transition (readiness for the transfer of care, developing self-advocacy skills), continuity and breadth of care (changing relationships, culture shock, new responsibilities), need for support (social support, mental health support, and ongoing support needs – beyond the transfer of care. Peer support was a connecting concept in the support sub-themes. Transition was more than a change in primary physician but also a change in the care model and breadth of care provided, which was challenging for young people especially if they had insufficient information.

**Conclusions:**

Transition from pediatric to adult care in rheumatology is a significant period for young people living with JIA, and this patient-led study provided insight into the experience from the perspective of young people with JIA which is critical to informing the development of supports for patients through the process. Patients, caregivers, pediatric and adult rheumatologists and members of the multi-disciplinary care team need to collaborate in terms of resources preparing for transfer, and support throughout the transition process to ensure a successful transition process.

**Supplementary Information:**

The online version contains supplementary material available at 10.1186/s41927-022-00316-5.

## Background

Juvenile idiopathic Arthritis (JIA) is a childhood autoimmune disease that causes swelling and pain in at least one joint. JIA is a common type of arthritis in children and teenagers, it affects approximately 3 in every 1000 Canadian children[1]. Some forms of JIA go into remission quickly and never return; others develop into a chronic condition that will follow the child into adulthood[2, 3]. Those that still have symptoms during their adolescent years will have to transfer from seeing their pediatric rheumatologist to seeing an adult rheumatologist. Transition includes a purposeful and planned transfer of teenagers and young adults from a pediatric-centre to an adult-centred care [4–7]. The transition process includes a period of preparation during adolescence, the event of transferring care, and continues after the event of transfer into adult care, reflecting the continued developmental stages into the mid-twenties and the need for developmentally appropriate rheumatology care [8] Transition from pediatric to adult care in rheumatology is a significant period, and transfer is a rite of passage for young people living with JIA. It can entail various stressors such as actual transfer in the delivery of care and increased responsibility for the young person with JIA in their patient role, and often coincides as well with the time of ‘emerging adulthood positioned between adolescence and young adulthood [9]. This period of life entails many significant developmental transitions such as moving out from family home, starting post-secondary education, or starting employment.

Current research on JIA transition emphasises clinical models of transition and health outcomes during transition [10–14]. Researchers have called for more studies on post-transition outcomes for individuals who have participated in different models of healthcare transition [13]. Young adults who have transferred from their pediatric relationship but continue to deal with challenges as an adult patient, are at greater risk of poor long-term outcomes[13]. A survey of adolescents and young adults at a single centre in the U.S., identified that few participants had received comprehensive counselling about the transition to adult care [15]. A study in Europe, where a clinical transition pathway was implemented found improvements in patient care, low drop-outs, high satisfaction and self-reported self-efficacy [16]. Understanding this transition process from the patient perspective is critical to ensure a successful process, and ultimately to achieve best long-term outcomes.

A recent systematic review of qualitative studies on the experience of transition for patients with juvenile-onset rheumatic conditions including JIA identified 26 studies from the United States, United Kingdom, Australia, Belgium, Canada, Denmark, the Netherlands, New Zealand, Norway, Ireland and Switzerland [17]. Key themes included preparedness for change, abandonment and fear of the unknown, sense of belonging, feeling anonymous and dismissed in adult care, quest for autonomy, and tensions with parental involvement. Challenges of transition identified include changing roles and tensions between wanting independence for the patient while still allowing caregiver involvement, organizational differences, and different service priorities and standards of care between child and adult health care with the adult environment perceived as daunting and resulting in feelings of isolation. Being informed about the transition process, coordination and preparation were reported as essential elements of successful transitions. There were two papers from the Canadian context identified in systematic review [18, 19] that were focussed on self-management in JIA and not explicitly about the transition process. Missing from the literature is research that centres the experience of emerging adults with JIA as they transition from pediatric to adult rheumatology care in Canada. This paper addresses this gap using a novel patient-engaged research approach.

A specific type of patient-engaged research methodology is the Patient and Community Engaged Research (PaCER) approach which seeks to engage, empower, and improve the lives of patient peers and community [20–22]. Patient researchers are trained in the PaCER methodology and play an active role in all aspects of the research process. The goal of this patient-led research project was explore the experience of Canadian young people with juvenile idiopathic arthritis in transition from pediatric to adult care.

## Methods

### The Patient and Community Engagement Research (PaCER) model

**Fig. 1:**
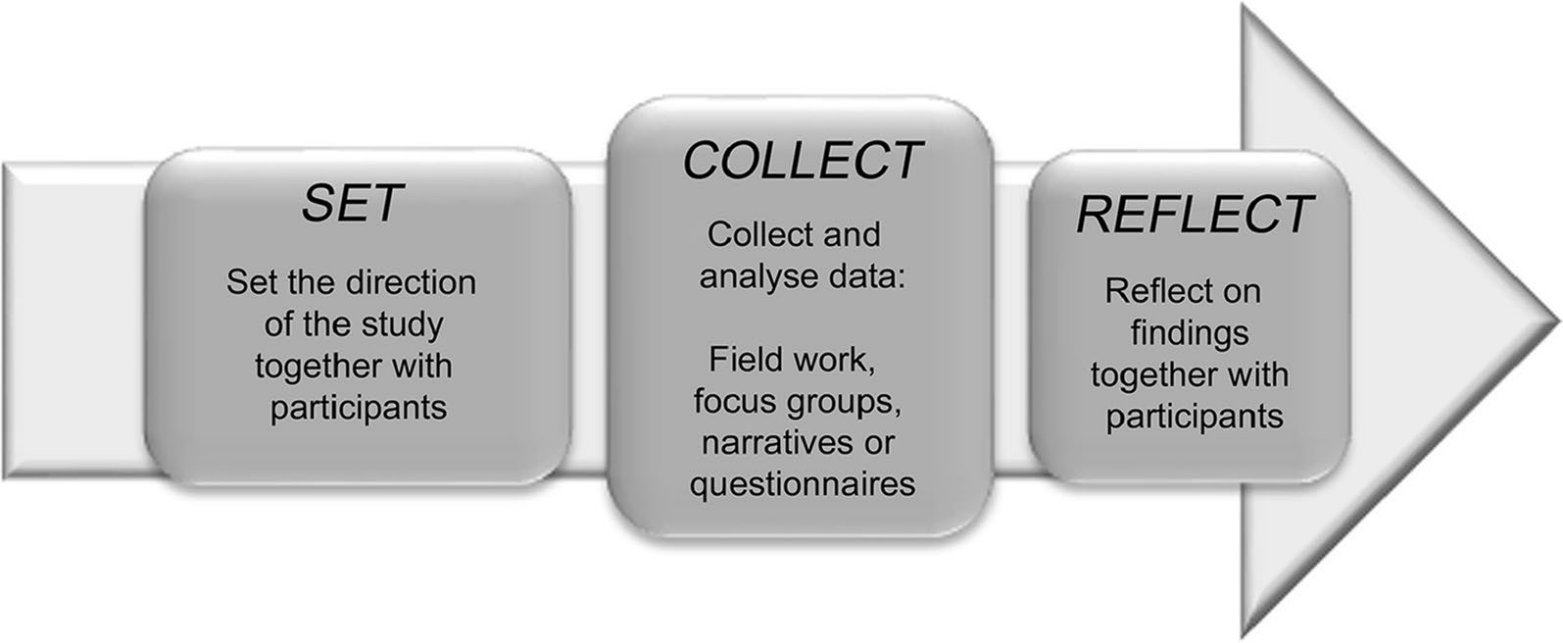
The PaCER research phases

A qualitative research design was used following the Patient and Community engagement Research (PaCER) approach [20–22]. PaCER has a distinct research framework that is designed to engage patients through three phases: Set, Collect, and Reflect (see Fig. 1 [20–23]). Patients are not only participants in the PaCER method, but also part of the research team. Five patient researchers (MH, LM, MS, NT,AvD), trained in the year-long PaCER program, were key research partners in research question development and they led the data collection and analysis process. In the Set phase, patient participants were invited to represent patient voices in a focus group to co-design with the patient researchers the topics of interest to prioritize what will best support patients. In Collect, qualitative data collection techniques (here focus groups) are used to collect and analyze data based on a specific topic based on results from the Set focus group. Participants are invited to share their own experiences. At this stage, the main ideas, themes, and patient concerns surrounding the topic can be identified. In the Reflect phase, participants from the Set and Collect focus groups, are invited to review the patient researcher’s interpretation of the collected data. Although transcripts are not returned to the participants, the PaCER team shares the preliminary study findings and Reflect participants are asked to give feedback on the themes identified. The Set and Reflect focus groups are hallmarks of the PaCER approach and ensure meaningful patient engagement and contextual validity.

### Identification of focus group participants: inclusion criteria and recruitment

Three focus groups (Set, Collect and Reflect) were conducted with patients who satisfied the inclusion criteria: aged 18–28 years old; spoke English; had self-identified as having a JIA diagnosis and who were in the process of transition (currently in process of transfer or had transferred) and were receiving treatment in Canada. Convenience and snowball sampling were to recruitment participants. Participants were recruited primarily through the University of Calgary’s Participate in Research page [24], through social media networks of the research team (including Take a Pain Check [25] and Rheumours [26]) and through a digital poster shared to the Cassie and Friends network [27]. Additionally, the research team shared the opportunity within their personal and social media networks which reached followers including casual acquaintances, co-workers, and classmates. Participants provided their consent to participate and provided demographic information via online surveys using Qualtrics.

### Data collection

The focus groups were approximately two hours in length and held over an institutional secure University of Calgary Zoom account. Focus groups were conducted using a semi-structured focus group guide (see Additional file 1) which was developed primarily based on the lived experience of the PaCERS, with guidance from the research team. The guide was not pilot tested. The interview process was iterative and responsive to the information collected in the focus groups. The patient researchers introduced themselves to the participants as fellow patients, and shared information about their own transition journeys. Two of the PaCERs served as facilitators (MH, LM) and guided the focus groups using prompts to gain a deeper understanding of participants’ experiences. The other PaCERS (MS, NT, AvD, TK) took field and process notes. There were no non-participants present on the online zoom call. The Set focus group was used to identify the topics that were most important to patient participant partners and to refine the questions that guided the Collect focus group. Participants at the Collect focus group were asked to describe what their lives were like during their transitions, and discussed their transition experience. Although there was no return of transcripts, the Reflect participants were presented the themes that had been identified from the analysis of the Collect data, asked to rank each theme’s importance in relation to their own transition experience. This ranking data was not collected for quantitative use, but rather to facilitate discussion of the themes to ascertain resonance and relevance of the themes. For each theme, a facilitated discussion enabled the participants to categorise and re-organize the data. Focus groups were recorded over Zoom and the audio recordings were transcribed verbatim. Transcriptions, in addition to field and process notes taken during the focus groups, were used in data analysis.

### Analysis

Thematic analysis was used to identify, analyze and report patterns (themes) within the data [28]. Qualitative analysis software was not used to code the data, but Excel was used to organize the coding. Data from the focus groups, process notes and field notes were coded. All PaCER researchers individually coded overlapping sections of the data, codes were then compared between researchers and themes were derived. Themes were then presented to participants at the Reflect focus group and ranked, and further refined. Analysis was complete when inductive thematic saturation was achieved based on the identification of no new codes and themes in the data collected [29]. All the codes identified were grouped into the identified themes.

### Reflexivity

The Patient and Community Engagement Research year-long program is delivered by the Department of Continuing Education and run through the AbSPOR Support Unit at the University of Calgary [30]. All the patient researchers (MH, LM, MS, NT, AvD) in this project were trained by this program. The patient researchers, all female, live with juvenile idiopathic arthritis, and ranged in age from 18 to 25 years old. The patient researchers of this study are from across Canada (Alberta, Ontario, and Saskatchewan). They have varying experiences with the transition from pediatric to adult care. Of the five PaCER researchers at the time the research was conducted, three had experienced the transfer of care from pediatric to adult care, and two were still in pediatric care but had experience of transitional care. During the conduct of this project, four of the patient researchers were in health related undergraduate education programs (psychology, nutrition, biomedical sciences, health studies), and one was employed in the health administration sector. One of the patient researchers (MS) had previous undergraduate research experience in the area of medical education. Another (NT) had experience as a patient partner on previous research, but no experience in conducting research. The other patient researchers (AvD, MH, LM) did not have prior research experience. The respective lived experiences of each individual researcher was fundamental in shaping both the collection and analysis of data for this project.

### Ethics

This study was approved by the University of Calgary Conjoint Health Research Ethics Board (REB-21-1326). The manuscript is reported according to consolidated criteria for reporting qualitative research (COREQ) [31].

## Results

### Sample characteristics

Nine participants were part of the one or more of the Set, Collect and Reflect focus groups. Five participants were part of the Set focus group. There were four participants at the Collect focus group including one who had taken part in the Set focus group. There were four participants at the Reflect focus group including three from the Collect group. No participants refused to participate or dropped out of the study. No demographic data was collected for the Set focus group, other than that all were female, between the ages of 18–28, and had either experienced or were experiencing the transfer to adult care. For the Collect and Reflect, all participants identified as female. Participants self-identified as white [4] and West Asian [1]. The age range of participants was 18–26 years old, with mean age of 22. Two had been diagnosed with JIA at under 3 years of age, two were diagnosed between 4 and 6 years old, one each were diagnosed between 10 and 12 years and 13–15 years of age. Two had a University degree, two had completed some University, and one had a diploma from a trade school or college. All participants were from an urban centre. Participants received care in the provinces of British Columbia, Alberta, Saskatchewan, and Ontario. Two participants received adult care in a different province than where they had received pediatric care. One participant was preparing for transfer of care via attending a formal transition clinic, but had not yet attended that clinic, and the others had not experienced care in a formal transition clinic.

### Analysis

**Fig. 2:**
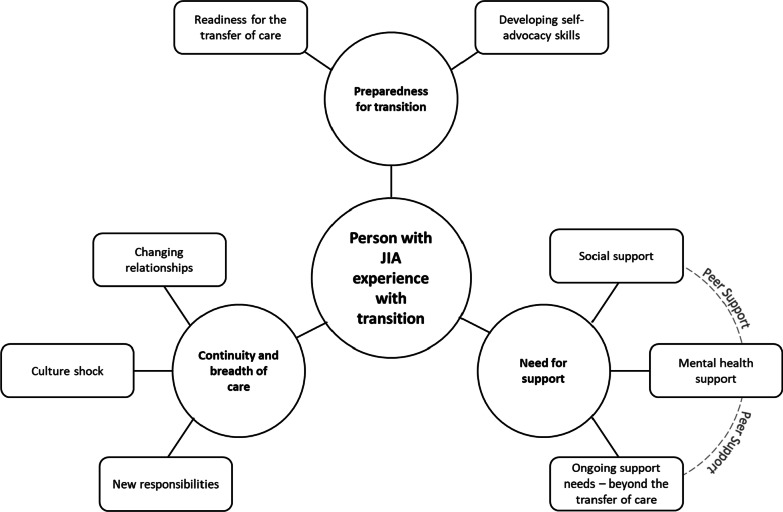
The experience of transition: themes and sub-themes

There were three themes and eight sub-themes that emerged from the data analysis. These themes and sub-themes are: Preparedness for transition (Readiness for the transfer of care, Developing self-advocacy skills), Continuity and breadth of care (Changing relationships, Culture shock, New responsibilities, and Need for Support (Social support, Mental health support, Ongoing support needs—beyond the transfer of care). Peer support is a connecting concept between the support sub-themes. See Fig. 2 for a representation of these themes, and their associated sub-themes. Table 1 shows representative quotes for each theme.

**Table 1:** Representative quotes for themes and sub-themes

Theme: Preparedness for transition	
Sub-theme	Quotes
Readiness for the transfer of care	“We went through… a checklist of… my medical history and my medication… she kinda helped me prepare… how the visits would… look different… the population area would be different.” (Participant 2) “By the age of 16, she wanted me to come in alone and… it kinda helped me just be more independent in that area.“ (Participant 2) “Just feeling used to doctors’ appointments and,… talking to doctors and being around nurses and all of that, like I just felt that kind of prepared me.“ (Participant 1) “I did feel prepared, I felt super prepared, and then, she completely changed it [the plan], and, and sprung it up, and then I had to put it all back together and convince them to let me go to the other place [Transition clinic vs Adult Rheum office].“ (Participant 5) “I consider myself extremely lucky… I took part in a research study when I was still in pediatric care… one of the biggest modules was self advocacy… So when I went into adult care, I felt that I had a lot of tools already… in my tool belt, so to speak… like, making lists before you go in. That you make sure everything gets checked off on your list and not leaving until you have done everything you need to do and have got all your medication, and all that stuff. So, um, I considered that probably one of the greatest resources I have had in my pediatric care.” (Participant 3) “When it came towards… the end it was… really upsetting and also… I was really anxious, because like, I wasn’t prepared to be in… a whole new environment… it kind of felt like starting from… scratch, like from the start with a whole new doctor. So I was kind of like nervous and stuff.” (Participant 2)
Developing self-advocacy skills	“The things you need to transition are learned skills, they’re not skills that come naturally. And so, of course, if that’s the case, you have to.“ (Participant 3) “I participated in… an online course on JIA and self-advocacy… This was the greatest resource I had in pediatric care.” (Participant 3) “I like to know what… my own responsibility should be when it comes to being transitioned into care. My pediatric rheumatologist was really helpful with that… We went through… a checklist of my medical history and my medication… [She] helped me prepare for how the visits would kinda look different and how the [waiting room] population area would be different.” (Participant 2)

Theme: Continuity and Breadth of Care	
Sub-theme	Quotes
Changing relationships	“I love my pediatric rheumatologist so much… she’s seriously just that awesome and I’ve known her since I was… three. So, it was a lot of bonding happening with her and I, so having to [say] goodbye to a doctor, but also, kind of a friend… it was hard.“ (Participant 4) “I loved my rheumatologist; she was really nice and [so was] the nurse there and we would always bond, and she would always make it such a safe environment. So, I [was] always comfortable to tell her what was wrong and everything else. But then, when it came towards the end, it was really upsetting… I was really anxious, because I wasn’t prepared to be in a whole new environment… it kind of felt like starting from scratch.” (Participant 2) “I’ve only seen this new doc - my new rheumatologist like, four to five times, because I usually go every six months. I haven’t been able to really establish a trusting, safe environment with him where we can have… similar conversations of how I would have with my previous rheumatologist.“ (Participant 2)
Culture shock	“I think it was a really stressful time. It would have been a bit nice to… talk to other kids going through the same things. Going from seeing a lot of kids to adults in their 50s and 60s was really hard, because I was 18… It was a big pill to swallow.“ (Participant 4) “You went from being surrounded by kids to being the youngest one there surrounded by like adults who are in their like, 50 and 60s. So that was definitely different.” (Participant 2) “I feel as though changing the structure which the pediatric appointments are conducted in rapidly is not a good… Is not good for the patient. Um, I think following a similar structure in terms of length of appointment and, um, how it is conducted, I think would provide a more seamless transition.” (Participant 5)
New responsibilities	“A sudden drop-in support was very jarring because it just really added to all these new responsibilities. And, you know, it was all in one appointment and then you had to outsource and all that [allied health care], so I just think that if you had more… continuity of care, it would be less… shocking, like being tossed in a cold pool.“ (Participant 3) “It’s almost like, I went from a teenager to… a young adult with a part-time job that was basically just taking care of appointments and medications and… it takes a lot of time and energy out of [you].” (Participant 1) “My rheumatologist helped me get resources on [insurance policies]. It was eye-opening… I was not educated on this at all. It was difficult to navigate.” (Participant 4) “The fact that there isn’t… a care team, it is difficult. It’s almost like, I went from a teenager to a young adult with a part-time job that was basically just taking care of appointments and medications… It takes a lot of time and energy out of yourself to make sure that you’re getting proper care.” [Participant 1]

Theme: Need for Support	
Sub-theme	Quotes
Social support	“I think that the support of my family was super important in transition.” (Participant 5) “Transitioning can be a very… isolating experience if you don’t have… support.” (Participant 1) “I found… it’s easier just having somebody knowing what I’m feeling or… having my mom… at appointments.” (Participant 1) “… I did have my mom… at my first couple of adult rheumatologist appointments… I think that sort of helped… me being able to speak up… ‘cause at the next one where she wasn’t there and I was all alone, it was like you are the only one who can advocate… like you’re the only one there for yourself.” [Participant 1] “I talked to my mom about… the idea of… not being able to take… your parent or… a support person in, and she said that that was… something that was really stressful for her.” (Participant 1) “Just seeing… 10 kids in a room that all have arthritis… just feels kind of… uplifting. Like you’re just… validated, like you’re not alone anymore.” (Participant 1) “… if I was able to connect with other people who had arthritis.you won’t feel so alone and you have someone who can relate… ” (Participant 2) “One really good resource that I had was the disability services through the university… so that was… a good support and sort of, a backbone to… help me how to figure out what I can and can’t do and then support me with talking to profs, or.changing my schedule.” (Participant 1)
Mental Health Support	“I grew up always… being told… you can do anything, don’t let your arthritis stop you… I wish that I would have known more… , and [had]… been a bit more realistic about what my limitations were… so that I didn’t struggle so much [at the start of university].” (Participant 1) “Stress… really impacts… my arthritis… it can be definitely a contributor… in flares and that, … is part of mental health.” (Participant 5) “I’ve also had… a positive impact on my mental health with transitional care because I felt like I had… more of… a self-accepting journey… as I got older, and I… was able to… connect with other people who had arthritis.” (Participant 5) “There was this service called clinical health psychology. So, they were there to support you when talking about like transitioning or kind of the mental health things that go along with you just being in pain all the time… and… not having others your age to relate to. So that was a really good service. It was… a good way for me to ease into sort of, what counseling or therapy would be like. And then to be able to be comfortable talking to somebody about what I was feeling… not just on a pain level anymore.” (Participant 1)
Ongoing support needs—beyond the transfer of care	“My adult rheumatologists are basically there for… my biomarkers and… what… my blood tests look like… so they don’t check in as much… with mental health and… I’m not even sure how much [information about mental health] to bring up to my adult rheumatologists.” (Participant 3) “I think having a psychologist built into [a] transition clinic or adult [clinic]… would be great. And I think then more people would access those mental health resources.” (Participant 5) “The most beneficial would be… relationship and social support, especially with youth who are also dealing with this and are about to transition. In my personal experience, I have never seen a person my age in the waiting room [in adult care]. But, when I go to camps and other meetings, I [think], ‘where have you been my whole life?’… If [there were] more support groups… [it] would be beneficial for people to relate and connect with each other, and also share resources and support with each other.” (Participant 2) “I would argue that you can prepare to a certain degree… and then, it would be nice that when questions come up… you have something to [draw] on, um, for those resources. " (Participant 3)

#### Preparedness for transition

##### Readiness for transfer of care

The concept of preparedness was identified by all participants as a key component of the patient experience during transition. While some patients felt ready to leave the pediatric system, the majority expressed feeling unprepared to move into adult rheumatology care.

*“I consider myself extremely lucky… I took part in a research study when I was still in pediatric care… one of the biggest modules was self advocacy… So when I went into adult care, I felt that I had a lot of tools already… in my tool belt, so to speak. like, making lists before you go in. That you make sure everything gets checked off on your list and not leaving until you have done everything you need to do and have got all your medication, and all that stuff. So, um, I considered that probably one of the greatest resources I have had in my pediatric care.”* (Participant 3).

*“When it came towards… the end it was… really upsetting and also… I was really anxious, because like, I wasn’t prepared to be in… a whole new environment… it kind of felt like starting from… scratch, like from the start with a whole new doctor. So I was kind of like nervous and stuff.”* (Participant 2).

##### Developing self-advocacy skills

Skills development and access to relevant resources increased patients’ feelings of preparedness for transition. Support from pediatric physicians or other caregivers was paramount in the development of disease management skills. Positive support included coaching on the use of checklists for medication and medical history (bringing lists to the appointment to have information on hand and ensure you cover what you need to cover in appointments), online courses for JIA patients, and simple discussions of what to expect at the first adult appointment. Even discussions of the new waiting room environment helped one participant feel more prepared.

*“I like to know what… my own responsibility should be when it comes to being transitioned into care. My pediatric rheumatologist was really helpful with that… We went through… a checklist of my medical history and my medication… [She] helped me prepare for how the visits would kinda look different and how the [waiting room] population area would be different.”* (Participant 2).

A variety of resources were discussed during the focus groups. One participant described an online course: Teens Taking Charge, created by pediatric rheumatology experts across Canada [32] completed during their early adolescent years. Several different modules covered different aspects of transition. The participant described the self-advocacy module as “…* the greatest resource [they] had in pediatric care*.” (Participant 3) Many participants discussed how healthcare professionals such as pediatric rheumatologists and pharmacists provided them with support during transition. A participant described their experience receiving guidance, “…* it was eye-opening… I was not educated on this at all. It was difficult to navigate.*” (Participant 2) A crucial resource given to participants by their healthcare team was information about insurance policies. Pharmaceutical customer support programs also provided participants with more insurance-related resources. One participant described how their pediatric rheumatologist guided them towards seeing a clinical health psychologist. Clinical health psychology, and other mental health resources were believed to be important resources in transition as they can alleviate the psychological burden of transition. Seeing that transition often coincided with the beginning of post-secondary education, student accessibility services were agreed upon by participants as being an important resource.

However, it was clear that these supports were available to some, but not all focus group participants during transition. Self-advocacy skills such as care coordination and learned disease management skills like medical literacy were identified as necessary for patients when navigating adult care, but require time, practice, and energy to master.

#### Continuity and breadth of care

Continuity of care was about the ease of transitioning to a new rheumatologist and care environment. Breadth of care is the scope of care that patients received throughout their transition experience.

##### Relationships

Focus group participants highlighted the importance of the strong, longstanding relationships they had built with their pediatric rheumatologists. For many patients, their pediatric rheumatology team had played a pivotal role in their development, and participants mourned the loss of this relationship when they were required to see a new physician.

“*I love my pediatric rheumatologist so much… she’s seriously just that awesome and I’ve known her since I was… three. So, it was a lot of bonding happening with her and… so having to [say] goodbye to a doctor, but also, kind of a friend… it was hard.*” (Participant 4).

##### Culture shock

When moving into adult rheumatology care, patients described feelings of culture shock, especially early on in their transition. This feeling was exacerbated by stark differences in appointment and care structures between pediatric and adult rheumatology. Patients struggled with drastic changes in the office environment, the format of adult rheumatology appointments (shorter and less frequent), and challenges building trust and rapport with a new healthcare professional.

When visiting their pediatric rheumatologist, participants found comfort in waiting rooms with other patients their age. In the adult environment, however, participants mentioned feeling extremely isolated – keenly aware of the fact that they were often half the age of anyone else in the waiting room. One participant mentioned that their adult rheumatology clinic worked to book all JIA patients on the same days, which helped with feelings of isolation brought on by an older waiting room population.

*“Going from seeing… a lot of kids to adults in their 50s and 60s was really hard, because I was 18… It was just a stressful time, all in all, just how everything was going on. It was, it was a lot. It was a big pill to swallow.”* (Participant 4).

“*You went from being surrounded by kids to being the youngest one there surrounded by like adults who are in their… 50s and 60s. So that was definitely different.*” (Participant 2).

Once past the waiting room, the shortened length of adult appointments left patients feeling rushed and unable to fully discuss their concerns. Increased time between appointments further contributed to patients’ feelings of dissatisfaction. Shorter appointments, suddenly fewer and further between, left little time for patients to build the trust with their new rheumatologist that they so valued with their pediatric rheumatologists.

##### New responsibilities

The breadth of care, defined as the range of medical care that a patient receives for their JIA, was also an adjustment for patients. Participants identified that most of their pre-transition medical care was followed by their pediatric rheumatologists or rheumatology teams including allied health professionals and other medical specialists, even when care did not directly involve JIA or their inflamed joints. Following transition, participants highlighted a sharp reduction in the scope of care that they received from their rheumatology teams, pointing to less concern for their overall wellbeing, mental health, and willingness to discuss other health concerns and more focus on JIA in isolation of these broader impacts. Participants discussed frustration and feelings of being overwhelmed with the challenge of managing their own interdisciplinary care teams in the face of a new rheumatology framework that didn’t assist in the coordination of this care.

Taking on new responsibilities in adult care left some participants struggling with the transition, feeling as though they were thrown into cold water with no support.

*“A sudden drop in support was very jarring because it just really added to all these new responsibilities. And, you know, it was [previously] all in one appointment and then [now] you had to outsource and all that [allied health care], so I just think that if you had more… continuity of care, it would be less… shocking, like being tossed in a cold pool”* (Participant 3).

The time it takes to coordinate necessary health services was compared to starting a new job:

*“The fact that there isn’t… a care team, it is difficult. It’s almost like, I went from a teenager to a young adult with a part-time job that was basically just taking care of appointments and medications… It takes a lot of time and energy out of yourself to make sure that you’re getting proper care.”* (Participant 1).

#### Need for support

##### Social support

Social support is the experience of being cared for by friends, family and others when dealing with JIA-related challenges. This can improve mental health and overall quality of life. Participants expressed often feeling lonely and isolated.

“*… I did have my mom… at my first couple of adult rheumatologist appointments… I think that sort of helped… me being able to speak up… ‘cause at the next one where she wasn’t there and I was all alone, it was like you are the only one who can advocate… like you’re the only one there for yourself*.” (Participant 1).

When discussing the emotional and physical challenges of JIA, participants highlighted access to social support was a helpful resource that improved their self-esteem, reduced distress, and supported their self-advocacy skills development during transition. Strong relationships between peers, family, physician, and the patients were crucial. A robust social support system paved the way for more positive experiences throughout a patient’s transition.

Two participants reported that changes to their care during transition caused additional stress for their families, who wanted to be there for their children and provide them with support but felt unable to assist during a pivotal period of time.

Patients reported that compassionate social support validated their experiences during transition. Some examples of social support resources that were beneficial included disability services at university.

*“One really good resource that I had was the disability services through the university… so that was… a good support and sort of, a backbone to… help me how to figure out what I can and can’t do and then support me with talking to profs, or changing my schedule.”* (Participant 1).

They also noted that peer support from others would be invaluable:

*“… if I was able to connect with other people who had arthritis… you won’t feel so alone and you have someone who can relate… ” (*Participant 2*)*.

The creation of such resources pertaining to social support was recommended by the participants. For example, participants identified that increased one-on-one support from a social worker with knowledge of the transition process would be valuable to enable them to successfully navigate transition (e.g., understanding disability rights).

##### Mental health support

Mental health support was described by participants as the support for patients’ mental health before, during and after transfer. This included mental health and emotional support from family, friends, psychologists, and other mental health specialists. A psychologist at one patient’s University aided them in coping with the feelings associated with having a chronic childhood disease, instead of solely focusing on the physical symptoms of the disease like at a rheumatology appointment. Multiple patients agreed that parental support for mental health surrounding transition came in the form of validation of their JIA, its symptoms and the difficulty of transitioning.

Receiving support during this time of transition in a young person’s life could make a difference in a patient’s JIA transition experience. There were many uncertainties in the patient’s personal lives, such as moving away from home, starting new jobs or starting post-secondary education. These external factors had an impact on mental health. Contributing factors to mental health struggles that patients faced included: anxiety induced by transitioning into adult care (specifically regarding the new clinic environment), trying to develop a sense of trust in the new care team and the feeling of not being cared for.

Mental health support needs were different across patients. Some patients found that transition did not have a negative impact on their mental health. One patient described their transition experience as a journey to self-acceptance with JIA:

*“it’s also had… a positive impact on my mental health… Before… there was… a lot of… stigma… especially with… kids…. But then as I got older… was able to connect with other people who had arthritis, I was able to… be more… self-accepting of it, and I feel like transitional care played a piece in that.”* (Participant 2).

Participants identified that stress or emotional challenges were a reliable trigger for their JIA flares. Patients found success with support from other people in their lives or other mental health professionals.

*“Stress… really impacts… my arthritis… it can be definitely a contributor… in flares and that,… is part of mental health.”* (Participant 5).

##### Ongoing needs: beyond transfer of care

Resources and support to understand aspects of the transition process were described by participants as being instrumental to a successful transition. There were differences reported regarding available resources for transition from pediatric to adult care by the participants. Participants who had access to resources and support found them to be highly useful during transition. Those who did not have access expressed that resources and support over time throughout the process would have improved their transition experience.

Participants agreed that there was a need for increased mental health resources and support during transition. Accessing these resources proved to be difficult. Following transition, participants found that mental health resources were not integrated as a key aspect of adult rheumatology care. Patients had to advocate and seek these resources independently. Ideally, these resources should be readily accessible for the JIA patient during transition. Participants expressed that it was unclear where to get mental health support. In their pediatric care, pediatric rheumatologists would check in on the mental health and wellbeing of patients. But it was not a priority or a topic of discussion amongst most patients seeing adult rheumatologists.

“*My adult rheumatologists are basically there for… my biomarkers and… what… my blood tests look like… so they don’t check in as much… with mental health and… I’m not even sure how much [information about mental health] to bring up to my adult rheumatologists… *” (Participant 3).

Another participant suggested:

*“I think having a psychologist built into [a] transition clinic or adult [clinic]… would be great. And I think then more people would access those mental health resources.”* (Participant 5).

Additionally, participants discussed a strong desire for increased peer-support during transition in the adult care environment. One participant said:

“*I have never seen a person my age in the waiting room [in adult care]. But, when I go to camps and other meetings, I [think], ‘where have you been my whole life’?*” (Participant 5).

## Discussion

This patient-led study provided insight into the transition experience from the patient perspective which is critical to informing the development of supports for patients through the process. Themes and sub-themes identified had support as well in previous qualitative studies as synthesized in a 2020 systematic review which included 16 studies. Preparedness for transition [17, 33], readiness for transfer of care [17], developing self-advocacy skills [17], continuity and breadth of care [17, 33], changing relationships [17], culture shock [17], new responsibilities [17], need for support [17], social support [17, 34], while additional emphases were identified by this study on mental health support and ongoing support needs – beyond the transfer of care. The Kelly et al. systematic review [17] identified support for the whole person (theme of deprived of human focus) which encompasses psychological support, but did not explicitly identify mental health support [17]. The idea of support beyond transfer of care was also touched on in the security of a reliable point of contact theme[17], however this study identified further depth in this theme area.

**Fig. 3:**
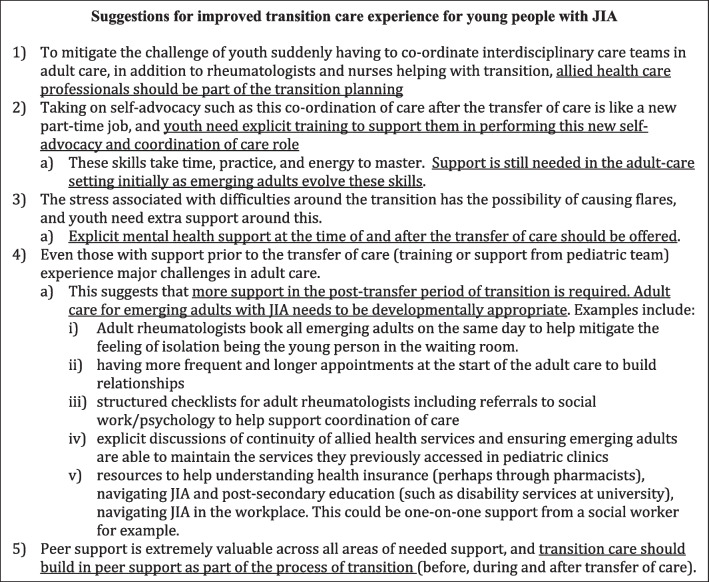
Suggestions for improved transition care experience for young people with JIA

Suggestions to improve transition care experiences for young people with JIA are summarized in Fig. 3.

A common sentiment included the value of social support to participants during their transition journey, especially when they were able to connect with other JIA patients. This support helped increase their self-confidence and self-compassion. Many participants did not feel ready for transition. If initially prepared, this could change by an unpleasant or unexpected encounter in the new environment. This stresses how important it is that patients are provided tools that help them feel prepared before but also to feel empowered throughout transition. This finding resonates with previous work which identified that the promotion of health self-efficacy and meeting the adult provider prior to transfer were features of care that predicted successful transitional care outcomes [35].

Of similar importance is strong, clear communication with healthcare providers as they play a large role in a patient’s transitioning journey. A healthcare provider has much power in affecting a patient’s stress levels, confidence, and self-perception during their transition from pediatric to adult care. When there is a significant change in breadth of care provided and little continuity between clinicians such as physiotherapists, occupational therapists and rheumatologists, patients may be confused as to who to turn to when looking for information.

Transition-related challenges can cause stress for patients, which can in turn impact their health. Participants identified that stress or emotional challenges were a reliable trigger for their JIA flares. While patients felt that pediatric rheumatology placed emphasis on mental wellbeing as an important aspect of their JIA management, they expressed frustration over a lack of attention to mental health when dealing with their adult rheumatologists. Other qualitative studies identified the importance of emphasizing managing daily life not just clinical outcomes [36, 37]. The the importance of attending to mental health needs of JIA patients is a key finding that resonates with other studies which have identified this gap in care [38, 39] It was not identified in other qualitative studies about transition however, and we hypothesize that the patient-to-patient research process may have facilitated participants to open up about this differently when speaking with fellow patients instead of with a traditional research facilitator.

These findings present a patient-centred view that is consistent with recently identified priorities for successful rheumatology transition care in Canada [14]. The priorities highlighted by Canadian rheumatologists are confirmed by our findings including calls for increased allied health support, access to mental health resources, and greater preparation for the adult medical system. However, while current literature on transition care in rheumatology from both patient and physician lenses [4, 14] assert the importance of successful patient integration into the adult healthcare system, further action is needed to tangibly improve the quality and accessibility of transition care in Canada. The findings also resonate with the recommendations of the Canadian Pediatric Society position statement for transition to adult care for youth with complex health care needs which include early transition planning, and support before and after transition [40]. Many of the themes also resonate with a qualitative study of mental health services transition in Canada, including the information needs in preparedness for transition, the changing roles and responsibilities for coordinating care, and the gaps in continuity of care [41].

The patient experiences shared in this study offer a unique glimpse into the lives of young adults as they navigate two vastly different healthcare systems. This research supports past qualitative findings of significant differences in the model and scope of care between pediatric and adult medical systems that have been identified in other countries [17, 33, 37]. Our findings indicate that this remains an issue in the Canadian context. Many existing transition initiatives work to support patients before and during their transition to the adult medical system. Current priorities emphasise the importance of transition readiness and patient education [14]. However, despite participating in transition preparation initiatives, and best-efforts on the behalf of their pediatric care teams, patients often still felt unsupported by their receiving rheumatologist post-transition. This discrepancy in patient experience suggests further ongoing support is required post-transition as patients adjust to care practices in adult rheumatology. In particular, there is a need to explicitly attend to the third stage of transition which occurs post-transfer in emerging adulthood, and a need for developmentally appropriate care in adult settings to ensure the success of the transition process [8].

As patients continue to struggle through a pivotal time in their young lives, this study reinforces the need for developmentally appropriate, easily accessible, direct-to-patient resources and increased support at all stages of the transition journey.

## Strengths and limitations

One of the challenges of this study included virtual focus groups held entirely on zoom. The focus groups were two hours in length and zoom fatigue became apparent nearing the end of the sessions. Had the focus group been in person, the participants would have had a chance to have snacks or refreshments. They likely would have felt more engaged for the second half of the focus group, which could have impacted our results. Additionally, in-person sessions, with a reasonable break, could have been longer and provided more participant data. Another limitation of Zoom focus groups was the difficulty in capturing and recording participants body language because of partial visibility. If body language could have been recorded our data could have been richer. On the other hand, the use of Zoom did enable the participation of individuals from across the geography of Canada with varying systems for transition, rather than limited to one location. It also increases accessibility for participants for whom travel to an in-person focus group setting could be more fatiguing than participation from their own home. Our sample lacked diversity. The participant sample only included female participants, thus lacked any unique perspectives that male participants may have added. Of note, male gender is a significant risk factor for care gaps [42]. No rural residents participated in the study, thus the experience of these patients is not reflected. It is likely that the transition experience of rural residents has particular challenges that would not be reflected here. None of the participants involved in focus groups had completed transition at a designated transition clinic, therefore that specific experience of transition was not represented. The availability of transition clinics varies across the country with some urban cities having rheumatology transition clinics, while other cities do not [11]. Future research should examine the experiences of a more diverse participant group.

The study had a relatively small sample size with nine participants. Saturation was assessed in the analysis of the data collected (inductive thematic saturation) rather than in the sampling (data saturation) [29]. Although the relatively small number of participants was sufficient to identify a range of thematic issues, a larger sample size would be needed to achieve a more richly textured understanding of the themes [43]. Comparing to existing literature, only one study in Denmark was smaller [6]. The themes do resonate with prior similar research providing some external validation. However, further research should focus on data saturation in a more diverse population to capture the experience of males, those who took part in a formal transition program, and those from rural and remote areas.

This study had unique strengths, first and foremost that the research was patient oriented and patient-led research using the PaCER methodology which is unique in its approach. The patient researchers co-defined the research question within the broad area of transitions in care. The process of data collection (with the three phases of Set, Collect, Reflect) and the process of analysis is led by patients which creates a space that truly centres the patient experience by enabling more meaningful engagement for the patient participants and increased contextual validity overall.

## Conclusion

The transition from pediatric to adult care is a significant event for JIA patients. Contributing to a successful transition were adequate resources and skills in preparation for transition, supports including social support, mental health support, and ongoing support in adult care, as well as efforts to support continuity of care especially in light of the change in breadth of care provided after transfer of care.

Future studies should include participants that had experienced a process that included a dedicated transition clinic, especially since in the past decade, more of these clinics have become available across Canada. To ensure successful transition experiences, it is important to include the involvement of the pediatric care team and adult care teams with patients and their family at all stages of the transition (prior, during and after transfer). Transition was more than a change in physician (the transfer of care), but also a change in the care model and breadth of care provided, which was challenging and abrupt for patients especially if they had insufficient information about what was to come. Patients, caregivers, pediatric and adult rheumatologists, and members of the multi-disciplinary care team need to collaborate in terms of resources and support for the transition process to ensure a successful transition experience for the patient before, during and after transfer of care.

## Supplementary Information

**Additional file 1:** Focus group guides.

## Data Availability

Under the terms of the ethics approval and consent, we are not able to share the raw focus group data for this qualitative study. Upon reasonable request to the corresponding author, we can provide some de-identified data.

## Abbreviations

JIA: Juvenile idiopathic arthritis
PaCER: Patient and Community Engagement Research
