# Supplementary material for: Transitions from pediatric to adult rheumatology care for juvenile idiopathic arthritis: a patient led qualitative study
Source: BMC Rheumatol. 2022 Nov 14;6:85. doi: 10.1186/s41927-022-00316-5 (PMC9664794; doi:10.1186/s41927-022-00316-5)
Supplement: Supplementary file 1 — Additional file 1: Focus group guides. [file 41927_2022_316_MOESM1_ESM.docx]

This supplementary files contains the focus group guide for the SET, COLLECT and REFLECT focus groups.

**JIA SET Focus Group Guide**

| **Item** | Time |
| --- | --- |
| **Introduction**   - Thank people for coming - Opening remarks + what is PaCER? - **Briefly explain the PaCER program and your training** - **Year-long training program** - **Patient-led** - **What is Co-design and the PaCER methodology** - Conducting focus group   - Go around the table and have everyone introduce themselves and explain the roles they will be assuming for the session - Brief intro of us all of us (name, age, where we from)   - Lauren, Kayla, Morgan, Alejandra, Natasha, Manahil, Tatiana - **Briefly explain the PaCER program and your training**   - **Year-long training program**   - **Patient-led**   - **What is Co-design and the PaCER methodology** - Establish some ground rules for your conversation | 4pm – 4:30pm MST |
| **Give a brief research summary (what have you done so far to inform your research question?)** | 4:30 – 4:45 pm MST |
| - Have you transitioned? What experience do you have with transition? Where are you from? - ICE BREAKER   - Allow the participants to introduce themselves Ice breaker question: What is 1 highlight of your month? (positive) Why are you interested in transition care? - Remind everyone that the purpose of the meeting is to help develop your research projects | 4:45– 5:15 MST |
| **Questions**   - - If you were doing this research project what would you study?   - When thinking about transition, what do you think is working really well?   - What could be improved?   BREAK   - - Do you have any suggestions of individuals or organizations we should speak with?   - Do you have any questions for us? | 5:15 – 6:30 PM MST |
| **Thank you!** | 6:45pm |

**Exploring the Patient Experience of Transition in Juvenile Idiopathic Arthritis (JIA)**

**COLLECT Focus Group Guide**

| **Time (will vary)** | **Activity** |
| --- | --- |
| 5 minutes | Welcome, Introductions, Administrative   - Welcome - Thank people for coming - Brief intro of us all of us (name, age, where we from)   1. Lauren, Morgan, Alejandra, Natasha, Manahil, Tatiana - Briefly explain the PaCER program and your training   1. Year-long training program   2. Patient-led   3. What is Co-design and the PaCER methodology - Establish some ground rules for your conversation - **Make sure your zoom name is your full name (first and last)** - Explain that each team member is completing a different role (notetaking) (WHO IS DOING WHAT) - Remind participants they can withdraw |
| 5 minutes | Purpose of this focus group:   - To discuss and explore the experiences of patients with Juvenile Idiopathic Arthritis in transition from pediatric to adult medical care |
| 5 minutes | Brief Research Summary   - We will be covering how health determinants impacted our journeys - Our experiences when it comes to transitions and the fears/positives behind it - Using academic literature and research papers - The SET focus group helped us to identify the direction of our research   These were the SET focus group highlights:   - Resource & support availability - Barriers & facilitators during the transition experience - Quality of life & mental health during transition - The Focus group will be recorded - START RECORDING |
| 10 minutes | ICEBREAKER   - Allow the participants to introduce themselves - Ice breaker questions:   - What is 1 highlight of your month? (positive)   - Why are you interested in transition care? |
| 30 minutes | Research begins by sharing:   - Describe what your life was like during your transition?   - How does your viewpoint on quality of life align with that of your medical care team?   - What was challenging?   - What was beneficial? |
| 5 minutes | BREAK |
| 30 minutes | - How did transition impact your quality of life and your mental wellbeing? (self-esteem, self-efficacy, self-advocacy, etc)   - - How did your self-advocacy evolve during your transition? - Can you recall any resources that have (or would have?) eased your transition?   - How has the availability of resources and support during transition affected your patient experience? - Is there anything else you would like to share about your transition experience that we have not covered today? |
| 10 minutes | BREAK |
| 15 minutes | Summarize the information collected (flip chart information).  *Discussion may include:*   - Variety of experiences - Common themes in regard to the experience of transitioning from pediatric to adult care |
| 10 minutes  Total: 2 hours | Wrap up and validation of new discoveries through engagement:   - - - Tell us what you discovered today through this group conversation?     - What will you take away from this session?     - Please come to our next FG we will be likely be holding it in the first weekend in November     - STOP RECORDING |

**Exploring the Patient Experience of Transition in Juvenile Idiopathic Arthritis (JIA)**

**REFLECT Focus Group Guide**

| **Time (will vary)** | **Activity** |
| --- | --- |
| 7 minutes | Welcome, Introductions, Administrative   - Welcome - Thank people for coming - Brief intro of us all of us (name, age, where we from)   - Lauren, Morgan, Alejandra, Natasha, Manahil, Tatiana - Briefly explain the PaCER program and your training   - Year-long training program   - Patient-led   - What is Co-design and the PaCER methodology - Establish some ground rules for your conversation - Remind participants they can withdraw |
| 3 minutes | Purpose of this focus group:   - The purpose of this focus group is to review the findings from the COLLECT and to discuss opportunities for dissemination and future research   - Review/analyze and rank our findings |
| 5 minutes | Brief Research Summary   - Our experiences when it comes to transitions and the fears/positives behind it - Using academic literature and research papers - The SET focus group helped us to identify the direction of our research - The COLLECT focus group allowed us to better understand the individual experiences of our patient participants |
| 10 minutes | RECORDING STARTS  ICEBREAKER   - Allow the participants to introduce themselves - Ice breaker questions:   - What was one win/highlight this month?   - What sparked your interest in joining us today? |
|  |  |
| 15 minutes | Summarize the information collected (flip chart information).  *Discussion may include:*   - Variety of experiences - Common themes regarding the experience of transitioning from pediatric to adult care |
| 30 minutes | - Does this information resonate with you?   - What resonates most with your experience of transition? - Ranking/prioritization activity:   - When thinking about the experience of transition, how important is the theme of NAME OF THEME     - Most relevant     - Moderately relevant     - Less relevant - Is there anything else that you feel is important to add that may have been missed? - Does this data answer our research question? |
| 10 minutes | BREAK |
| 20-30 minutes | - Recap today’s discussion/prioritized themes - How should our findings be shared? - Is there an opportunity for future research here? - Is there anything else you would like to share about your transition experience that we have not covered today? |
| 5-10 minutes  Total: 2 hours | Thank you for coming!  If time allows:  Wrap up and validation of new discoveries through engagement:   - - - Tell us what you discovered today through this group conversation?     - What will you take away from this session? |
